# [18F]FDG PET/MRI of patients with chronic pain alters management: early experience

**DOI:** 10.1186/2197-7364-2-S1-A84

**Published:** 2015-05-18

**Authors:** Sandip Biswal, Deepak Behera, Dae Hyun Yoon, Dawn Holley, Ma Agnes Martinez Ith, Ian Carroll, Matthew Smuck, Brian Hargreaves

**Affiliations:** Stanford University School of Medicine, California, USA

The chronic pain sufferer is currently faced with a lack of objective tools to identify the source of their pain. The overarching goal is to develop clinical [18F]FDG PET/MRI methods to more accurately localize sites of increased neuronal and muscular metabolism or inflammation as it relates to neurogenic sources of pain and to ultimately improve outcomes of chronic pain sufferers. The aims are to 1) correlate imaging findings with location of pain symptomology, 2) predict location of symptoms based on imaging findings alone and 3) to determine whether the imaging results affect current management decisions. Six patients suffering from chronic lower extremity neuropathic pain (4 complex regional pain syndrome, 1 chronic sciatica and 1 neuropathic pain) have been imaged with a PET/MRI system (time-of-flight PET; 3.0T bore) from mid thorax through the feet. All patients underwent PET/MR imaging one hour after a injection of 10mCi [18F]FDG. Two radiologists evaluated PET/MR images (one blinded and the other unblinded to patient exam/history). ROI analysis showed focal increased [18F]FDG uptake in affected nerves and muscle (approx 2-4 times more) over background tissue in various regions of the body in 5 of 6 patients at the site of greatest pain symptoms and other areas of the body (SUVmax of Target 0.9-4.2 vs. Background 0.2-1.2). The radiologist blind to the patient history/exam was able to correctly identify side/location of the symptoms in 5 out of 6 patients. Imaging results were reviewed with the referring physician, who then determined whether a modification in the management plan was needed: 1/6 no change, 2/6 mild modification (e.g., additional diagnostic test ordered) and 3/6 significant modification.

